# Energy and Transmission Efficiency Enhancement in Passive Optical Network Enabled Reconfigurable Fronthaul Supporting Smart Homes

**DOI:** 10.3390/s20216245

**Published:** 2020-11-02

**Authors:** Rentao Gu, Gang Wang, Zhekang Li, Yuefeng Ji

**Affiliations:** Beijing Key Laboratory of Network System Architecture and Convergence, School of Information and Communication Engineering, Beijing University of Posts and Telecommunications, Beijing 100876, China; bestwanggang@bupt.edu.cn (G.W.); zhekangli@bupt.edu.cn (Z.L.); jyf@bupt.edu.cn (Y.J.)

**Keywords:** mobile networks, network algorithms, network resources allocation, cloud radio access networks

## Abstract

Smart home technologies are growing actively all around the world. As a result, great pressures are imposed on internet of things networks by dynamic traffic and plenty of devices. The passive optical network is considered one of the most promising fronthaul technologies. In particular, the time and wavelength division multiplexing passive optical network has shown the advantage of high capacity and received attention recently. In support of internet of things networks, the energy and transmission efficiency has emerged as an important issue on the time and wavelength division multiplexing passive optical network enabled fronthaul networks. In this paper, we try to enhance the energy and transmission efficiency of the time and wavelength division multiplexing passive optical network enabled reconfigurable fronthaul. Fronthaul links’ load balancing is also taken into consideration. An integer non-linear programming model is employed to formulate the joint optimization problem. We also provide an adaptive genetic algorithm-based approach with fast convergence. The simulation results show that the active units of fronthaul can be dynamically switched on/off with the traffic variation and a significant energy saving is achieved. In addition, the maximum transmission efficiency increases by 87% with integer non-linear programming method in off-peak periods.

## 1. Introduction

The artificial intelligence and multimedia enabled smart home significantly facilitates our daily life [1,2,3]. However, high dynamic and high peak-to-average ratio traffic is generated by the above services. The explosive growth of dynamic traffic imposes great pressure on internet of things (IoT) and related networks. Furthermore, Cisco’s research forecasted that billions of IoT connections will be added by 2023 [4]. The proliferation of diverse IoT devices may change the design of traditional IoT networks [5]. In smart homes, sensors (including the video surveillance system) are connected to the gateway through short distance network connections, such as Wi-Fi, ZigBee, and NB-IoT [6]. In the 5G era, the information collected by the gateway can be transmitted to cloud via 5G cellular mobile networks [6] and is stored and analyzed in cloud servers [7]. To transmit signals, the network between the baseband units (BBU) and remote radio units (RRUs) is called mobile fronthaul network in the centralized radio access network (C-RAN) architecture [8,9]. Then, the mobile fronthaul network will be a key factor to support various smart home services [10], which is the first network segment to connect smart homes. However, facing the dynamic and busty traffic of smart homes, the energy and transmission efficiency has emerged as an important issue on fronthaul networks.

Considering the progressive network evolution, the 5G non-standalone (NSA) will be widely deployed in the first stage, in which cloud radio access network (C-RAN) is still being used [11]. Thus, in this paper, we will take C-RAN based fronthaul as a typical scenario during the analysis; nevertheless, the proposed algorithms can be easily extended for 5G standalone (SA), since the topology between active antenna unit (AAU) and distributed unit (DU) is similar to that between RRUs and BBUs.

The energy and transmission efficiency problems should be taken into consideration. In C-RAN, the central BBUs are responsible for coordinated signal processing based on high-performance processors. The RRUs are located at target areas to serve the access equipment [12]. The digital signal transmission between centralized BBUs and distributed RRUs is based on the low-latency and high-capacity passive optical network (PON). Based on current noted radio interface (e.g., common public radio interface (CPRI) [13], e-CPRI [14], next generation fronthaul interfaces (NGFI) [14]), oversampled in-phase/quadrature-phase (I/Q) signals are transferred via capacity-constrained fronthaul network. The transmission efficiency of the optical fronthaul is badly affected, since the CPRI bitrate is constant, independent of the traffic fluctuation [15].

Research on energy efficiency of C-RAN has been widely explored [16,17,18,19,20,21,22,23,24,25,26,27,28,29,30,31,32,33,34,35,36,37,38,39,40,41] from different perspectives, including resource allocation in BBU pool, function splitting, power control in upstream/downstream, etc. However, little attention has been focused on the energy and transmission efficiency of the reconfigurable optical fronthaul, especially for the passive optical networks based fronthaul system. In our previous works, we have discussed the efficient resource allocation algorithms for time and wavelength division multiplexing passive optical network (TWDM-PON) enabled fronthaul under different scenarios [42,43,44,45], but without complete energy consumption consideration.

The explosive growth of high dynamic traffic imposes great pressure on internet of things and related networks. As the first network segment to connect smart homes, the mobile fronthaul network faces the dynamic and busty traffic of smart homes. The energy and transmission efficiency has emerged as an important issue on fronthaul networks. As the strict requirement of green communication, how to achieve an energy-efficient configuration becomes more important.

In this paper, we address the energy and transmission efficiency enhancement problem of fronthaul network to support smart home networks. Moreover, the load balancing of the fronthaul is discussed and analyzed. Specifically, we first present a quantitative study of the problem using integer non-linear programming (INLP) model. Afterwards, an adaptive genetic algorithm (GA) based approach is proposed to reduce the complexity of the INLP model. Finally, we present numerical simulation analysis. This paper gives an in-depth detailed study of a joint optimization, including energy saving and transmission efficiency enhancement problem of optical fronthaul. Load balancing in the fronthaul links is also taken into consideration.

The rest of the paper is organized as follows. Section 3 formulates the INLP model, the adaptive GA is described in Section 4, and the simulation results are discussed in Section 5. Finally, Section 6 summarizes the results.

## 2. Related Works

Research on the energy efficiency of C-RAN has been widely explored from different perspectives, including resource allocation in the BBU pool, function splitting, power control in upstream/downstream, etc.

Wang et.al. proposed an efficient energy saving approach by implementing virtual base station. Each cell will be assigned to virtualized network resources [16]. Liu et al. developed a network energy consumption model for H-CRAN [17]. In addition, Fiorani et al. analyzed the viable fully centralized LTE radio architectures [18]. Alhumaima et al. proposed a power model to dynamically adjust the active virtual machines in BBU pool [19]. The model evaluated the processed resource blocks (RBs). Latency imposed by virtualization, minimum data rate of mobile equipment, and the number of RBs have been considered in the model. Deep learning was used to model and predict the energy efficient mapping between baseband processing tasks and micro-servers [20]. An efficient BBU and RRU association algorithm was investigated on the basis of graph partitioning and rejoining [21]. The power consumption was significantly reduced by this algorithm. Further, two efficient schemes were proposed to improve both the power savings and survivability of the network by using optical ring network in C-RAN [22]. And novel bounds on the user rate function were proposed [23]. The system energy efficiency was maximized by optimizing the transmit powers while explicitly incorporating the capacity constraints on fronthaul. Ahmad et al. addressed the problem of energy efficiency maximization in C-RAN under the constraints of fronthaul capacity and per-BS transmit power [24]. Machine learning approaches are also considered in various scenarios; for example, AI-driven autonomous optical network architectures were proposed [25,26,27,28,29], and the architectures provided power self-adaptive capability according to the network condition.

Further, different fronthaul functional splits have been evaluated concerning the energy consumption of C-RAN [30]. In distributed antenna systems of C-RAN, considering the power constraints of the users, energy efficiency can be improved by designing the transmit precoding matrices [31]. Liu et al. redesigned the signal quantization method to maximize the system throughput in orthogonal frequency division multiple access (OFDMA) based C-RAN [32]. The wireless power control mechanism was taken into consideration in the joint optimization also. Zhou et al. formulated a joint problem to minimize the aggregate power consumption [33]. The problem contains RRH mode selection, precoding design, and fronthaul compression.

The tradeoff between queuing delay and energy efficiency was analyzed under the Lyapunov framework with power allocation and interference constraints [34]. Meanwhile, Zhao et al. proposed a cluster content caching architecture for C-RAN [35]. Two distributed algorithms were investigated to enhance the energy efficiency of the network and quality of service (QoS) experience of the mobile equipment under the architecture. Moreover, mobile cloud computing (MCC) technique was implemented in C-RAN [36]. A convex problem was formulated to joint energy minimization in the BBU pool with MCC. The problem took the constraints of tasks into consider (e.g., computing capacity, and transmitting power). Moreover, two compression approaches were introduced to maximize the downlink throughout of C-RAN over ergodic fading channels [37]. The compression technique was dependent on the functional split in the physical layer. The performance of the compression technique was influenced by the channel state information overhead caused by the large-scale of C-RAN. Li et al. proposed a novel bandwidth allocation algorithm and adjusted the upstream optical network unit (ONU) order [38]. The algorithm provided efficient low jitter upstream transmission in PON. Recently, machine learning techniques have been used for improving the performance of networks [39]. And a smart collaborative automation (SCA) scheme was designed to improve resource usage and overcome buffer limitations [40]. Luong et al. designed transmit beamforming, remote radio head (RRH) selection, and RRH-user association jointly to maximize energy efficiency [41].

## 3. System Model

In this section, the considered TWDM-PON enabled smart home fronthaul network is introduced. Then joint energy and transmission efficiency problem of the optical fronthaul is formulated using INLP optimization method.

### 3.1. TWDM-PON Enabled C-RAN Architecture

**Figure 1:**
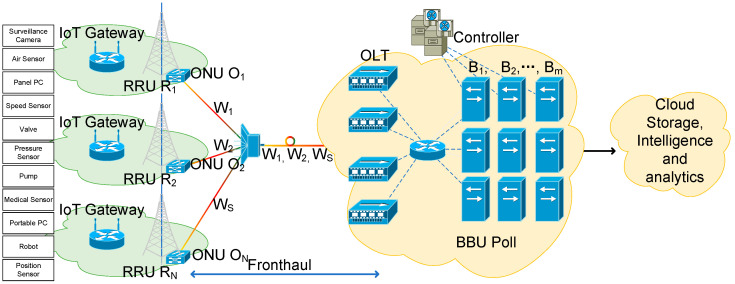
Architecture of IoT network in 5G NSA.

Dedicated point-to-point fibers is a solution candidate for fronthaul transport network. However, considering of the greenhouse effects and energy shortage, it is not attractive due to the high cost and energy consumption. The energy consumption mainly caused by the large-scale deployment of fibers and transceivers. PON is gaining more attention because of its great potential [46,47,48]. Different PON techniques have been widely explored for fronthaul, such as wavelength division multiplexing (WDM) PON [46,47], and subcarrier multiplexing (SCM) PON [48]. Meanwhile, TWDM-PON is regarded as a potential solution candidate for the fronthaul, because of its high capacity and low energy consumption [49]. Figure 1 depicts the energy efficient reconfigurable TWDM-PON enabled C-RAN architecture for smart home network. In C-RAN, ubiquitous RRUs perform the radio signals forwarding and digitizing from all mobile devices in each small cell. We assume that only one corresponding ONU is connected with each RRU serving the smart home gateway. Multiple CPRI links are de-multiplexed/multiplexed at the optical line terminal (OLT). In the BBU pool, baseband digital signals are processed by the virtualized BBU, based on the real-time flexible scheduling and high-performance processors.

For the reconfigurable fronthaul network, TWDM-PON is a promising candidate solution, whose benefits has been widely explored [49]. The traffic from multiple ONUs can be multiplexed in the same wavelength resource, thanks to the time and wavelength multiplexing technique. Different independent virtual PONs (VPONs) will be formed when different wavelengths are used for data transmission [20], leading to a reconfigurable virtual fronthaul network. In addition, it is known that the tidal effect is obvious during the day in both office and residential cells. However, in traditional C-RAN, the CPRI bitrate is constant regardless of traffic variation, while dependent of the number of used antennas. For instance, for one 2 × 2 multiple input multiple output (MIMO) and 20MHz bandwidth LTE sector, the required CPRI link rate is 2.4576 Gbit/s, leading to a low transmission efficiency since only 150 Mbit/s is useful for the mobile devices in a small cell on the air interface [50]. To accommodate the high demand traffic, different fronthaul compression techniques have been widely studied [37,51]. Meanwhile, compression technique based bitrate-variable CPRI was introduced [42]. This paper considers compressed bitrate-variable CPRI as a key factor for the challenge in smart home. Furthermore, based on the operation mode of each small cell, part of the fronthaul links and other active units such as ONUs and transceivers can be shut down without energy supply at proper times [52,53,54]. Moreover, both virtual topology of reconfigurable fronthaul and operation modes of small cells can be abstracted in the controller based on the software defined network technique. Energy and transmission efficiency enhancement approaches can also be easily implemented and updated with the traffic variation during the days.

### 3.2. Problem Formulation

Consider a TWDM-PON enabled C-RAN system with M BBUs denoted by $B_{1}$, …, $B_{M}$, and N RRUs denoted by $R_{1}$, …, $R_{N}$. The mobile devices in the small cells communicate with the ubiquitous distributed RRUs, and the RRUs are connected to the centralized BBUs through a reconfigurable TWDM-PON enabled fronthaul. We consider that there are N ONUs denoted as $O_{1}$,…, $O_{N}$, deployed in the proposed C-RAN. Each ONU is co-located with the corresponding RRU in the target area. Meanwhile, let $W_{1}$,…, $W_{S}$ represent the set of wavelengths used in the reconfigurable fronthaul. Each wavelength is served by a transceiver in the OLT, which is co-located with the BBU pool.

In TWDM-PON, an active transceiver consumes a constant energy $P_{Tr}$. ONU and RRU consume a constant energy $P_{ONU}$, and $P_{RRU}$, independently, when both of them are active. Integer N indicates the number of ONUs or RRUs at remote small cells. Integer S indicates the number of transceivers in the OLT. Boolean variables $s_{i}$, $r_{i}$, and $\sigma_{j}$ indicate whether the ith ONU, RRU, and jth transceiver are active, respectively. The capacity of a wavelength is 10 Gbit/s, denoted as $C_{W}$. Boolean variable $\delta_{Wj,\left(O_{i},R_{i}\right)}$ indicates that whether wavelength Wj is distributed to ONU $O_{i}$ (or RRU $R_{i}$) for signal forwarding. Parameter $\nu_{CPRI}$ represents the link rate of CPRI. The variable $\mu_{Ri}$ indicates the compression ratio of RRU $R_{i}$ (or ONU $O_{i}$) on the basis of bandwidth requirement in the corresponding small cell.

In this section, we only study the energy saving of the fronthaul, since the energy consumption of BBU pool has been widely explored in previous works [16,17,18,19,20,21,22,23,24,25,26,27,28,29,30,31,32,33,34,35,36,37,38,39,40,41]. Active networks devices, except the active equipment in the BBU pool, including transceivers, ONUs, and RRUs. An active transceiver consumes a constant energy PTr. When it is shut off, no energy is consumed. Similarly, the operation mode of the ONU and RRU varies with the smart home traffic fluctuation. They consume a constant energy $P_{ONU}$, and $P_{RRU}$, independently, when both of them are active. Otherwise, no energy is consumed when they are in sleep mode. So, energy consumption for the fronthaul with optimization is:

(1) $$P_{fronthaul}=\sum_{i=1}^{N}\left(P_{ONU}\cdot s_{i}+P_{RRU}\cdot r_{i}\right)+\sum_{j=1}^{S}P_{Tr}\cdot \sigma_{j}$$

Integer N indicates the number of ONUs or RRUs at remote small cells, and integer S indicates the number of transceivers in the OLT. Moreover, Boolean variables $s_{i}$, $r_{i}$, and $\sigma_{j}$ indicate whether the ith ONU, RRU, and jth transceiver are active, respectively. Specifically, $s_{i},\;or\;r_{i},\;o_{r}\;\sigma_{j}=1$ means that the corresponding device is active. Otherwise, the active network device is shut off.

For traditional TWDM-PON enabled fronthaul without energy minimization optimization, all transceivers, ONUs, and RRUs are always active, and the energy consumption is constant as follow:

(2) $$P_{fronthaul}^{’}=P_{Tr}\cdot S+\left(P_{ONU}+P_{RRU}\right)\cdot N$$

Furthermore, it is known that the traditional CPRI link rate is constant independent of traffic variation. Even low smart home flow may lead to a high bandwidth requirement and low transmission efficiency of the optical fronthaul. In this section, we also try to improve the transmission efficiency of TWDM-PON enabled reconfigurable fronthaul, on the basis of the compressed bitrate-variable CPRI [42]. In the proposed C-RAN, the capacity of a wavelength is 10 Gbit/s, denoted as $C_{W}$. Each wavelength is served by a transceiver in the OLT co-located with the BBU pool. So $\sigma_{j}=1$ can also indicate that the wavelength $W_{j}$ is used for data forwarding in the fronthaul optical links. The transmission efficiency of TWDM-PON enabled fronthaul is defined as follow:

(3) $$\eta =\frac{S-\sum_{j=1}^{S}\sigma_{j}}{S}$$

As we know, the tidal effect is clear during the day in both the smart homes of office and residential. Uneven requirements among RRUs results in load imbalance in fronthaul links. We take the load balancing in the wavelength dimensioning into consideration in the problem formulation. We use mean squared error (MSE) (similar to [42]) to indicate the load balancing state of the TWDM-PON enabled fronthaul network:

(4) $${LB}_{fronthaul}=\frac{\sum_{j=1}^{S}\sigma j\cdot {\left(\begin{array}{l} \sum_{i=1}^{N}r_{i}\cdot s_{i}\cdot \delta_{Wj},\left(O_{i},R_{i}\right)\cdot \nu_{CPRI}\cdot \mu_{Ri}\\ -\left(\sum_{i=1}^{N}r_{i}\cdot s_{i}\cdot \nu_{CPRI}\cdot \mu_{Ri}\right)/\sum_{j=1}^{S}\sigma_{j} \end{array}\right)}^{2}}{\sum_{j=1}^{S}\sigma_{j}}$$

where Boolean variable $\delta_{Wj},\left(O_{i},R_{i}\right)$ indicates that whether wavelength $W_{j}$ is distributed to ONU $O_{i}$ (or RRU $R_{i}$) for signal forwarding. Specifically, $\delta_{Wj},\left(O_{i},R_{i}\right)=1$ if $W_{j}$ is allocated to ONU $O_{i}$ (or RRU$R_{i}$), and 0 otherwise. $\left(O_{i},R_{i}\right)$ means that there is a corresponding ONU $O_{i}$ is connected with RRU $R_{i}$ serving the small cell. Moreover, parameter $\nu_{CPRI}$ represents the link rate of CPRI. The variable $\mu_{Ri}$ indicates the compression ratio of RRU $R_{i}$ (or ONU $O_{i}$) on the basis of bandwidth requirement in the corresponding small cell.

Our objective is to enhance the energy and transmission efficiency of the proposed TWDM-PON enabled reconfigurable fronthaul. Moreover, we also consider the load balancing between the optical fronthaul links. Three sub-objectives are given by Equations (1), (3) and (4), respectively. We use INLP approach to optimize the proposed three sub-objectives with the following constraints:

(5) $$s_{i}\in \left\{0,1\right\},\;r_{i}\in \left\{0,1\right\},\forall_{i}\in \left\{1,\ldots ,N\right\}$$

(6) $$\sigma_{j}\in \left\{0,1\right\},\;\delta_{Wj},\left(O_{i},R_{i}\right)\in \left\{0,1\right\},\forall_{i}\in \left\{1,\ldots ,N\right\},\forall_{j}\in \left\{1,\ldots ,S\right\}$$

(7) $$\sum_{i=1}^{N}s_{i}\cdot r_{i}\cdot \delta_{Wj},\left(O_{i},R_{i}\right)\cdot \nu_{CPRI}\cdot \mu_{Ri}\leq C_{W},\forall_{j}\in \left\{1,\ldots ,S\right\}$$

(8) $$\sum_{j=1}^{S}s_{i}\cdot r_{i}\cdot \delta_{Wj},\left(O_{i},R_{i}\right)=0,\;or\;1,\forall_{i}\in \left\{1,\ldots ,N\right\}$$

(9) $$\sum_{j=1}^{S}\sum_{i=1}^{N}\delta_{Wj},\left(O_{i},R_{i}\right)=\sum_{i=1}^{N}s_{i}\cdot r_{i}.$$

Value constraints of variables $s_{i}$, $r_{i}$, $\sigma_{i}$, and $\delta_{Wj,}(O_{i},R_{i})$ are shown in Equations (5) and (6). Equation (7) limits the maximum aggregated CPRI signal transmission rate served by an active wavelength $W_{j}$. Equation (8) indicates that only one wavelength can be assigned to each active ONU $O_{i}$ (or RRU $R_{i}$), and zero otherwise. Equation (9) ensures that the total number of active ONUs (RRUs) obtained by two different ways is the same with each other.

## 4. Adaptive Genetic Algorithm for Joint Optimization

In this section, we provide an adaptive GA based approach to achieve the near-optimal solutions for the joint optimization of energy and transmission efficiency of the reconfigurable fronthaul. We also consider the load balancing problem in the wavelength dimensioning.

### 4.1. Genetic Encoding and Evaluation

GA is a popular nature selection based heuristic approach to efficiently search the near-optimal solutions with low complexity [55]. The search procedures of GA are based on the mechanics of natural selection and natural genetics. GA is developed to allow computers to evolve solutions to difficult function optimization problems [56,57]. The basic operation of a GA could be divided into three steps: (1) maintain a population of solutions to a problem, (2) select the better solutions for recombination with each other, and (3) use their offspring to replace poorer solutions. A candidate solution for the given problem is encoded as a chromosome (or an individual). Each individual is evaluated by a fitness function, and better individuals have more opportunities to go through further genetic procedures: including selection, crossover, and mutation operations.

Each optical fronthaul link, including corresponding transceiver, ONU, and RRU, is indicated by a gene vector. The gene is encoded in $\zeta_{i,j}=\left\{s_{i},r_{i},\sigma_{j},\delta_{Wj},O_{i},R_{i}\right\}$. Boolean variables $s_{i}$, $r_{i}$, and $\sigma_{j}$ indicate whether the ith ONU, RRU, and jth transceiver are active, respectively. In addition, Boolean variable $\delta_{Wj},\left(O_{i},R_{i}\right)$ shows that whether wavelength $W_{j}$ is distributed to ONU $O_{i}$ (or RRU$R_{i}$) for signal forwarding.

For the energy and transmission efficiency optimization problem in the reconfigurable fronthaul, our objective is to search a series of near-optimal Boolean variables (e.g., $s_{i}$, $r_{i}$, $\sigma_{j}$, and $\delta_{Wj},\left(O_{i},R_{i}\right)$) that satisfied with Equations (1), (2) and (4). Each wavelength is served by a transceiver in the OLT, which means that there is one-to-one correspondence between wavelength $W_{j}$ and $\sigma_{j}$. A chromosome is consisted of N genes, denoted as $Ι=\left\{\zeta_{1,j1},\ldots ,\zeta_{N,jN},j_{1},\ldots ,j_{N}\in \left\{1,\ldots ,S\right\}\right\}$. Then we define a population with K individuals as ψ. Each individual is a candidate solution for the energy and transmission efficiency problem in the TWDM-PON enabled fronthaul.

The initial population ψ with K individuals is formed randomly. Since there are three sub-objectives in the problem, weighting method is introduced during optimization. The fitness function is shown in Equation (10).

(10) $$F\left(Ι\right)=\left(\alpha_{1},\alpha_{2},\alpha_{3}\right)\cdot {\left(\frac{1}{P_{fronthaul}},\eta ,\frac{1}{{LB}_{fronthaul}}\right)}^{’}\left(Ι\right)$$

Meanwhile, during the chromosome generation for initial population, selection, crossover, and mutation phase, not all individuals are feasible, since some of them may not meet the constraints shown in Equations (7)–(9). A penalty function is adopted to face the potential problem, considering of the constraints described in Section 4. For any illegal individual who does not satisfy the constraints, a penalty is added to decrease its fitness value. Finally, the modified fitness function is shown in Equation (11).

(11) $$F^{’}\left(Ι\right)=\{{}_{F\left(Ι\right)-\varepsilon 0\cdot \sum_{k=1}^{3}penalty(k,Ι),\;Ι\;is\;illegal}^{F\left(Ι\right),\;Ι\;is\;legal}$$

where ε0 is defined as the penalty factor, indicating the degree of penalty. Penalty function penalty(k,Ι) returns 0 if individual Ι meets the corresponding constraint, and 1 otherwise.

### 4.2. Adaptive Genetic Operations

Roulette wheel selection (RWS) is introduced in the implementation in selection phase. Selection probability *p*(I*_k_*) is assigned to each individual to implement weighted selection. The selection probability is proportional to individual’s fitness value. The probability of the individual Ιk is given by:

(12) $$p\left({Ι}_{k}\right)=\frac{F’\left({Ι}_{k}\right)}{\sum_{j=1}^{K}F’\left({Ι}_{j}\right)}$$

The proposed algorithm randomly generate a number P in the interval [0, 1], and the individual ${Ι}_{k}$ who meets the Equation (13) is chosen. The individuals with a higher weight probability have more opportunities to be selected. The RWS operator repeats K times to form a new population.

(13) $$k=\min\left\{s|\sum_{j=1}^{s-1}p\left({Ι}_{j}\right)\leq P,s\in \left\{1,\ldots ,K\right\}\right\}$$

Then the new population is transferred to the crossover phase. Crossover inherits the idea of natural reproduction. A crossover probability denoted as $\rho_{c}$ is applied for the offspring generation of the selected parents. In this phase, one-point crossover is used. We first randomly generate a number p in the interval [0, 1]. If $p\leq \rho_{c}$, then a crossover point in the range {1, …, N − 1} is selected randomly, and the pair of parents swap the genes at the crossover point to generate two new individuals. The offspring inherit parts of characteristics of their parents, and they are supposed to provide better candidate solutions for the joint energy and transmission efficiency optimization. K individuals are generated by this way.

After crossover, all individuals go through the mutation phase. New genetic characteristics can be introduced into the population in this stage. Unlike the crossover, mutation operator can only randomly modify the genes of an individual. A mutation probability denoted as $\rho_{m}$ is applied for gene modification. For every gene in each individual, we apply the following mutation procedure. First, we randomly generate a number p in the interval [0, 1]. If $p\leq \rho_{m}$, then that gene is replaced with another one. In each genetic operation phase, the size of generated population maintains constant.

To achieve a better performance of the proposed GA, an adaptive approach by dynamically updating $\rho_{c}$ and $\rho_{m}$ is adopted. We apply Equations (14) and (15) [58] to obtain the value of $\rho_{c}$ and $\rho_{m}$ in each iteration. Specifically, we have $F\max=\max j\left(F^{’}\left({Ι}_{j}\right)\right)$, $Fmean=\sum_{j=1}^{K}F^{’}({Ι}_{j})/K$, and $Fc=\max\left(F^{’}\left({Ι}_{j1}\right),F^{’}\left({Ι}_{j2}\right)\right)$. Moreover, $\lambda_{c}$ and $\lambda_{m}$ are the default constant value of crossover probability and mutation probability.

(14) $$\rho_{c}=\{\begin{array}{ll} \frac{F_{\max}-F_{c}}{F_{\max}-F_{mean}}, & F_{c}\leq F_{mean}\\ \lambda c, & otherwise \end{array}$$

(15) $$\rho_{m}=\{\begin{array}{ll} \frac{F_{\max}-F^{’}\left({Ι}_{j}\right)}{F_{\max}-F_{mean}}, & F^{’}\left({Ι}_{j}\right)\leq F_{mean}\\ \lambda m, & otherwise \end{array}$$

We apply Equation (16) [58] to evaluate the convergence performance of the proposed adaptive GA approach, where $d\left({Ι}_{j1},{Ι}_{j2}\right)$ shows the differences between two individuals ${Ι}_{j1}$ and ${Ι}_{j2}$. Based on the proof in [59], we can state that the proposed heuristic method based adaptive GA has been converged, if GA’s degree of diversity denoted as $D_{p}$ is lower than a threshold.

(16) $$D_{p}=\frac{2}{K\cdot \left(K-1\right)}\sum_{j1=1}^{K-1}\sum_{j2=j1+1}^{K}\frac{d\left(j1,j2\right)}{N}$$

## 5. Numerical Results

In previous sections, both thee INLP approach and heuristic method based adaptive GA are investigated to enhance the energy and transmission efficiency of the TWDM-PON enabled reconfigurable fronthaul. We also take the load balancing in the wavelength dimensioning into consideration. In this section, numerical simulation results are provided and analyzed, to evaluate the performance of the proposed algorithms.

**Figure 2:**
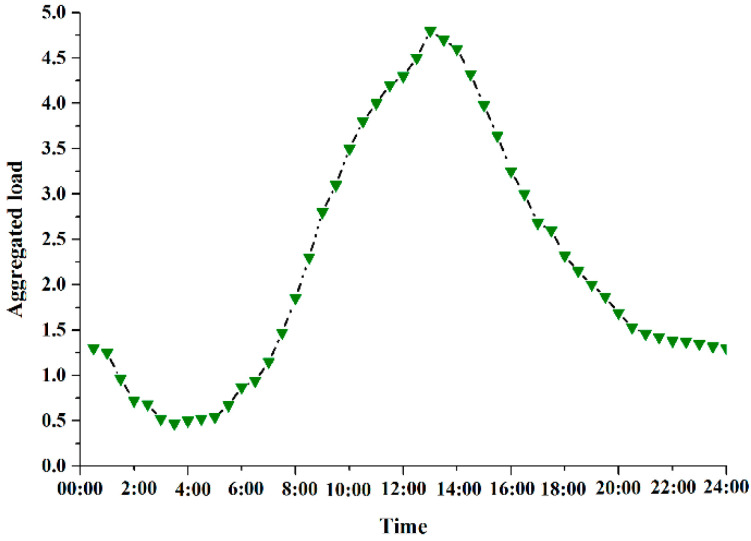
Traffic variation during an operational day [55].

We consider a TWDM-PON enabled C-RAN system with N = 32 RRUs, each served by a one-to-one corresponding ONU. And S = 8 wavelengths are assumed to be used in the fronthaul network, forwarding the compressed bitrate-variable CPRI signals [38]. Moreover, it is known that the tidal effect is clear during the day in smart homes of office and residential. We consider a business area, where the typical traffic variation during an operational day is shown in Figure 2 [60]. In order to reduce the response time, predicted traffic is used in the proposed algorithm. Other parameters are shown in Table 1.

**Figure 3:**
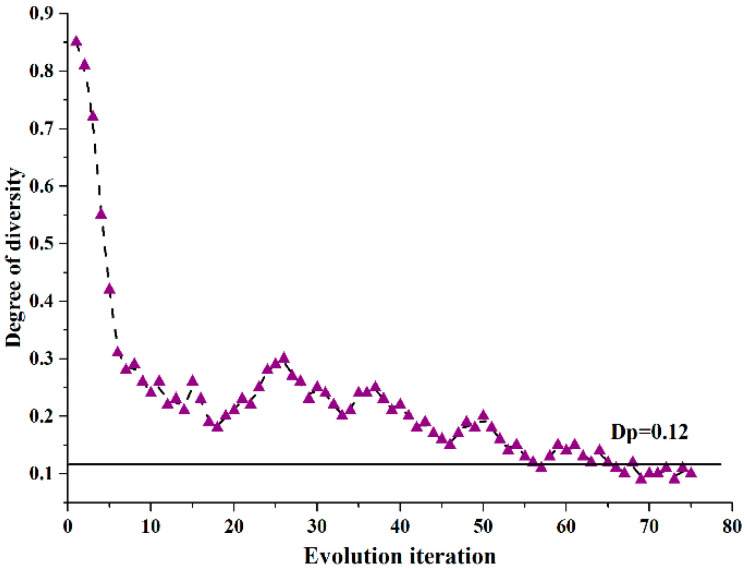
Convergence condition for proposed GA.

Figure 3 illustrates the heuristic method based adaptive GA’s converging condition. The initial population is consisted of a group of distributed individuals, each representing a candidate solution for the joint optimization. The diversity of the candidate solutions is increasing with the growing number of initial individuals. However, more hardware resources are required in the implementation. So, we find a proper population size K=100 by evaluating several population sizes. It is clear that when we take the degree of diversity Dp=0.12, and assume that the size of population K=100, the GA achieves the convergence within 65 iterations.

The running time of the adaptive GA is about 2.74 s, which is less than the traditional GA with fast convergence. Compared to the INLP approach, much lower running time is obtained by using the adaptive GA method on a computer with 3.2 GHz Intel Core 4 CPU and 4 GB RAM. This is because that the INLP method takes Million times iterations to achieve the global optimal solution. The adaptive GA is more efficient to find the near-optimal solution for the large-scale network optimization.

**Figure 4:**
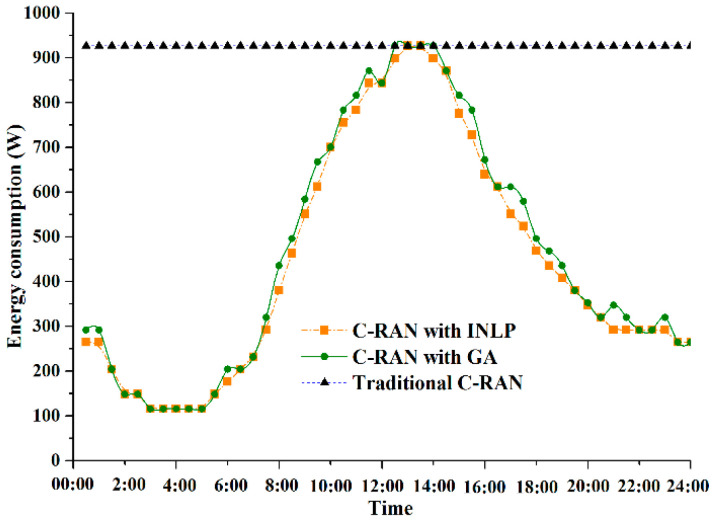
Energy consumptions of fronthaul with different methods.

Figure 4 presents the energy consumptions of TWDM-PON enabled reconfigurable fronthaul under three different conditions. Typical C-RAN with fixed topology and power supply consumes the most energy, since all the units including transceivers, ONUs, and RRUs are supposed to remain active, and supplied with energy all the time independent of traffic variation. C-RAN with INLP method obtains the global optimal solution for fronthaul energy minimization, leading to a significant energy saving. The active units of fronthaul network are dynamically turned off during appropriate times with the traffic fluctuation, whereas the INLP method is confronted with the lack of hardware resources. Moreover, C-RAN with the proposed adaptive GA approach achieves a better energy minimization solution than the C-RAN with fixed power supply. Compared to the INLP method, slightly less energy saving performance can be obtained using GA approach with lower complexity. By dynamically reconstructing the fronthaul virtual topology and adjusting the operation modes of the active units, energy efficiency of the fronthaul can be improved.

**Figure 5:**
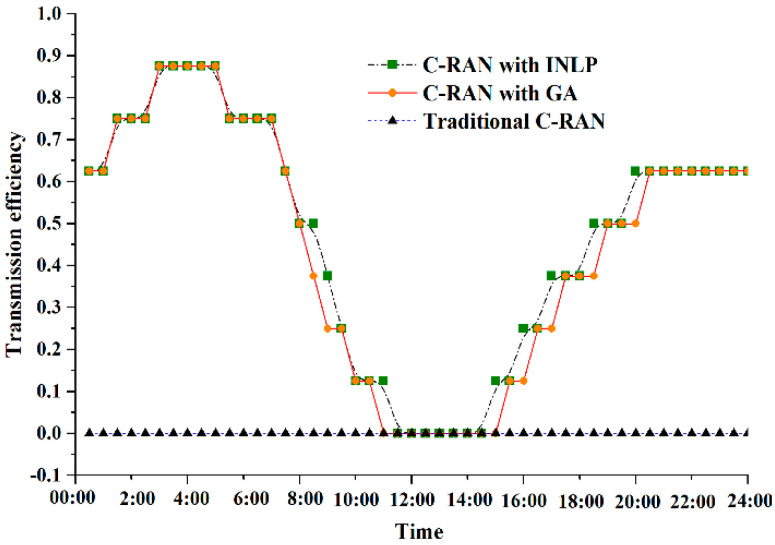
Transmission efficiency of fronthaul with different methods.

Figure 5 presents the transmission efficiency of fronthaul during an operational day with different joint optimization methods. In C-RAN with fixed topology connection relationship, all wavelength resources are occupied in forwarding CPRI signals regardless of load variation, leading to a significant bandwidth waste. Meanwhile, the corresponding transceivers are active all the time, which brings in additional energy consumption. Based on Equation (3), the transmission efficiency η is equal to zero in C-RAN with fixed topology, which is regarded as the benchmark. Moreover, global optimum can always be achieved by INLP method with exponential complexity. Wavelength resources are dynamically allocated based on the aggregated bandwidth requirements from each active ONU. The maximum transmission efficiency increases by about 87% with INLP method in off-peak periods. Whereas in peak time periods, the performance of INLP method is not obvious, since nearly all the wavelength resources are used to satisfy the bandwidth requirements. In addition, the proposed efficient GA achieves closely transmission efficiency enhancement as the INLP method, with linear complexity. Specifically, suboptimal solutions are obtained by adaptive GA, when traffic is decreasing or increasing during an operational day. This is because that there is a tradeoff between evolution times and optimization performance.

**Figure 6:**
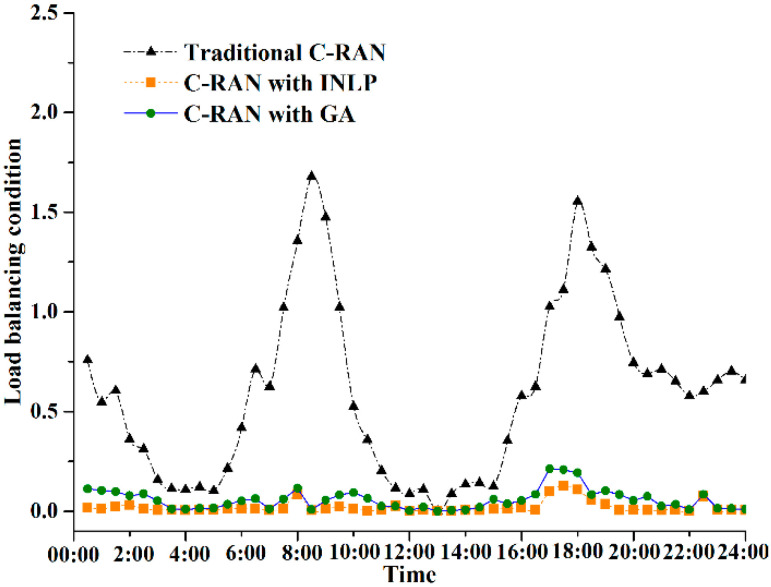
Load balancing conditions with different methods.

Figure 6 presents the load balancing performance of TWDM-PON enabled reconfigurable fronthaul, achieved by different approaches. The load balancing ${LB}_{fronthaul}$ is defined in Equation (4). As shown in Figure 2, it is observed that traffic changes over time during a day. In C-RAN with fixed wavelength resource allocation scheme, load imbalance is obvious when traffic is decreasing or increasing. Uneven requirements among RRUs results in load imbalance in fronthaul links. Meanwhile, in peak periods, nearly all the wavelength resources are on heavy load, and almost all the wavelengths are on light load in idle periods. Thus, the load imbalance is not clear in peak/idle periods. However, with the proposed INLP method and adaptive GA, the load imbalance of TWDM-PON enabled reconfigurable fronthaul is optimized independent of traffic variation during a day. This is because that the fronthaul network is dynamically reconstructed based on the amount of traffic served by each wavelength. This means that traffic is centralized in less wavelength resources, leading to a load balancing in the wavelength dimensioning.

## 6. Conclusions

This paper tried to study the energy and transmission efficiency problem of smart home supporting fronthaul transport network. The efficiency is improved by reconfigure the TWDM-PON. We also took the load balancing in the fronthaul links into consideration. Specifically, we considered employing an INLP model to formulate the joint optimization problem, which has exponential complexity. Afterwards we provided an adaptive GA based approach to reduce the complexity of the INLP method. The results showed that the active units of fronthaul including transceivers, ONUs, and RRUs could be dynamically switched on/off with the traffic variation by using the proposed GA, leading to a significant energy saving in TWDM-PON enabled fronthaul. Moreover, transmission efficiency has been improved with the joint optimization methods. The maximum transmission efficiency increased by about 87% with INLP method in off-peak periods. Moreover, with the proposed INLP method and adaptive GA, the fronthaul network was dynamically reconstructed based on the amount of traffic served by each fronthaul link. The load imbalance of TWDM-PON enabled reconfigurable fronthaul was further reduced independent of traffic variation during a day. Compared to the INLP method, almost the same performance could be achieved using an adaptive GA approach, with less complexity.

In the future, machine learning algorithm could be adopted for real-time prediction and response for better performance, such as artificial neural network and deep reinforcement learning. However, the vulnerability of machine learning should be considered to avoid unexpected reliability and security issues during network automation.

## Figures and Tables

**Table 1:** Simulation Parameters.

Simulation Parameters	
$P_{Tr}$, per-active transceiver consumption	5 W [61]
$P_{ONU}$, per-active ONU consumption	7.7 W [62]
$P_{RRU}$, per-active RRU consumption	20 W [20]
$C_{W}$, per-wavelength maximum capacity	10 Gbit/s [61]
$\nu_{CPRI}$, per-CPRI link rate (option 3)	2.5 Gbit/s
$\mu_{i}$, compression ratio for each ONU	0.2-1
K, constant population size for GA	100
$\rho_{c}$, initial crossover probability	0.8
$\rho_{m}$, initial mutation probability	0.05
$D_{p}$, convergence threshold for GA	0.12
$\left(\alpha_{1},\alpha_{2},\alpha_{3}\right)$	(1/2, 3/8, 1/8)
